# Evaluation of anti-diabetic potential of leaves and stem of *Flacourtia jangomas* in streptozotocin-induced diabetic rats

**DOI:** 10.4103/0253-7613.70238

**Published:** 2010-10

**Authors:** Ajay Kumar Singh, Jyoti Singh

**Affiliations:** Department of Pharmacology, School of Pharmaceutical Sciences, Jaipur National University, Jaipur 302 025, Rajasthan, India

**Keywords:** Antidiabetic activity, *Flacourtia jangomas*, hypogycemic, lipid profile, streptozotocin

## Abstract

**Objectives:**

To study the efficacy of combination of *Flacourtia jangomas* leaf and stem (1:1) methanolic extract (MEFJ) in streptozotocin (STZ)-induced diabetic rats and to investigate the qualitative phytochemical present in the extract. The study also aims to evaluate acute and short-term general toxicity of the extract in rats.

**Material and Methods:**

MEFJ of leaves and stem was subjected to preliminary qualitative phytochemical investigations by using standard procedures. The extract (400 mg/kg p.o.) was screened for antidiabetic activity in STZ-induced diabetic rats (30 mg/kg, i.p.). Acute oral toxicity study for the test extract of the plant was carried out using OECD/OCED guideline 425.

**Results:**

Phytochemical analysis of MEFJ of leaves and stem revealed the presence of flavonoids, saponins, carbohydrates, steroids, tannins, and phenolic compounds. In acute toxicity study, no toxic symptoms were observed for MEFJ up to dose 2000 mg/kg. Oral administration of MEFJ for 21 days exhibited highly significant (*P* < 0.01) hypoglycemic activity and also correction of altered biochemical parameters, namely cholesterol and triglycerides significantly (*P* < 0.05). Urine analysis on 1^st^ day showed the presence of glucose and traces of ketone in the entire group except normal control group. However, on 21^st^ day glucose and ketone traces were absent in MEFJ- and glibenclamide-treated groups while they were present in diabetic control. The data were analyzed using analysis of variance followed by Dunnett’s test.

**Conclusion:**

The observations confirm that methanolic extract of the leaf and stem of the plant has antidiabetic activity and is also involved in correction of altered biological parameters. It also warrants further investigation to isolate and identify the hypoglycemic principles in this plant so as to elucidate their mode of action.

## Introduction

Diabetes mellitus (DM) is a chronic metabolic disorder which affects a significant portion of the population worldwide.[1] DM is a group of metabolic diseases characterized by hyperglycemia, hypertriglyceridemia and hypercholesterolemia, resulting from defects in insulin secretion, its action or both.[2] Both type 1 and type 2 diabetes are known to be multifactorial diseases caused by a combination of genetic (inheritance) and environmental (diet and lifestyle) factors.[3] Non-insulindependent diabetes mellitus (NIDDM) is a multifactorial disease, which is characterised by hyperglycemia and lipoprotein abnormalities.[4] These traits are hypothesised to damage cell membranes, which results in excess generation of reactive oxygen species. NIDDM has also been associated with an increased risk for developing premature atherosclerosis due to an increase in triglycerides (TG) and low-density lipoproteins (LDL), and decrease in high-density lipoprotein levels (HDL).[5] Two groups of oral hypoglycemic drugs, sulphonylureas and biguanides, have been used in the treatment of DM. They act by lowering blood glucose thereby delaying or preventing the onset of diabetic complications.[6]

*Flacourtia jangomas* (family Flacourtiaceae) are large shrubs or small trees, 5–10 m tall.[7] Two limonoids, i.e. limolin and jangomolide were reported from the stem and bark of *F. jangomas*.[8] Leaves and bark are slightly acid and acrid and reported to be useful in diarrhea, piles, weakness of limbs, bleeding gums, toothache, and stomatitis.[9]

## Material and Methods

### Collection and authentication of plant

Leaves and stem of the plant were collected from local region of Kushinagar, District of Gorakhpur, India in the month of March 2009. The botanical identity was confirmed by a taxonomist Prof. Kamal, Department of Botany; Gorakhpur University, Gorakhpur where voucher specimen (No. GU0309186) has been deposited.

### Preparation of plant extract

The leaves and stem of *F. jangomas* were washed, shade dried and powdered. The powdered material was defatted with petroleum ether (60–80°C) and then extracted with methanol in Soxhlet apparatus (40 cycles). The extract was concentrated for further studies at reduced pressure and temperature in a rotary evaporator. MEFJ was tested for the presence of secondary metabolites by various phytochemical tests.

### Experimental animals

Healthy Wistar albino rats of either sex (150–200 g) were housed in polypropylene cages in an air-conditioned area at 25 ± 2°C with 12/12 h light–dark cycle. All animals had free access to standard pellet diet (Mahavir Industries, Delhi) and clean water *ad libitum*. The norms for Good Laboratory Practice (GLP) were followed for care of laboratory animals. The study was approved by Institutional Animal Ethical Committee (IAEC, clearance no: 003/2009/IAEC/jnu).

### Drugs and chemicals used

*Glibenclamide, streptozotocin (STZ)*, and sodium citrate buffer were used in this study. Other chemicals used for extraction purpose and phytochemical tests were of laboratory grade.

### Phytochemical screening

The plant may be considered as biosynthetic laboratory for the chemical compounds such as carbohydrates, protein, lipids, alkaloids, glycosides, tannins, etc. The compounds that are responsible for therapeutic effect are usually the secondary metabolites. A systematic study of a crude drug embraces thorough consideration of both primary and secondary metabolites derived as result of plant metabolism. The plant material may be subjected to preliminary phytochemical screening for detection of various plant constituents.

Standard screening test of the extract was carried out for various plant constituents. The crude extract was screened for the presence or absence of secondary metabolites such as alkaloids, carbohydrate, phenolic compounds, flavonoids, saponins, steroids, tannins, etc. by using standard procedures.[10,11]

### Acute toxicity test

Acute oral toxicity study for the test extract of the plant was carried out using OECD/OCED guideline 425. The test procedure minimizes the number of animals required to estimate the oral acute toxicity. The test also allows the observation of signs of toxicity and can also be used to identify chemicals that are likely to have low toxicity.

Healthy, young adult albino Wistar rats (200–250 g) were used for this study. Animals were fasted (food but not water was withheld overnight) prior to dosing. The fasted body weight of each animal was determined, and the dose was calculated according to the body weight.

### Limit test at 2000 mg/kg

The drug was administered in the dose of 2000 mg/kg body weight orally to one animal. This first test animal survived. Then, four other animals were dosed sequentially; therefore, a total of five animals were tested. Animals were observed individually at least once during the first 30 min after dosing, periodically during the first 24 h (with special attention given during the first 4 h), and daily thereafter, for a total of 14 days. No animal died. Therefore, the LD_50_ is greater than 2000 mg/kg.[12]

An investigation with 1/20^th^, 1/10^th^, and 1/5^th^ of 2000 mg/ kg, i.e. 100, 200, and 400 mg was done in pre-screening. Only 400 mg/kg was found to be effective against diabetes, hence this dose was used in final screening.

### Antidiabetic activity[13–15]

After fasting, DM was induced by intraperitoneal injection of STZ dissolved in 0.1 M cold sodium citrate buffer (pH 4.4) at a dose of 30 mg/kg b.w. The animals were allowed to drink 5% glucose solution overnight to overcome the drug-induced hypoglycemia. After 72 h, STZ-treated animals were considered as diabetic when the fasting plasma levels were observed above 200 mg/dL with glucosuria. The experiments were conducted on animal groups to see the effect of MEFJ on diabetic rats. Five rats were used in each of the four groups which were as follows:

Group I: Normal control (vehicle).
Group II: Diabetic control (vehicle).
Group III: Diabetic rats treated with MEFJ (400 mg/kg p.o.).
Group IV: Diabetic rats treated with glibenclamide (5 mg/kg p.o.).

Vehicle, MEFJ, and glibenclamide were administered once daily for 21 days from the day of induction. Blood was drawn from tip of the tail, and blood glucose level was estimated on 0, 7^th^, 14^th^, and 21^st^ day of experiment with the help of glucometer (one touch ultra, Johnson and Johnson Ltd.) using strip method. On 21^st^ day, blood sample was taken by orbital sinus bleeding method for measuring serum cholesterol and TG level using an auto-analyser (Semi auto chemistry analyser, CHEM 400). Fresh urine was collected, and glucose and ketone in urine were checked using keto-diastix strips on 0 and 21^st^ day of the experiment.

### Statistical analysis

All results were expressed as mean ± SEM. The data were analyzed using analysis of variance (ANOVA), and the group means were compared by Dunnett’s test. Values were considered statistically significant with *P* < 0.05. GraphPad Instat was used for the analysis of data.

## Results

### Preliminary phytochemical screening

Phytochemical screening was done using color forming and precipitating chemical reagents to generate preliminary data on the constituents of the plant extract. The chemical tests revealed the presence of major secondary metabolites such as flavonoids, carbohydrate, steroids, tannins and phenolic compounds, saponins, etc. in the extract of the leaves and stem of *F. jangomas*. The results indicated the presence of flavonoids, saponins and carbohydrate, steroids, tannins, and phenolic compounds in methanolic extract of *F. jangomas*.

### Acute toxicity studies

A preliminary toxicity study was designed to demonstrate the appropriate safe dose range that could be used for subsequent experiments rather than to provide complete toxicity data on the extract. Acute toxicity studies conducted revealed that the administration of methanolic extract (up to a dose of 2000 mg/kg) of *F. jangomas* did not produce significant changes in behavior of the animals. No death was observed up to the dose of 2000 mg/kg b.w. The rats were physically active. These effects were observed during the experimental period (14 days). The results showed that in single dose the plant extract had no adverse effect, indicating that the medium lethal dose (LD_50_) could be greater than 2000 mg/kg body weight in rats. In acute toxicity study, no toxic symptoms were observed for MEFJ up to dose of 2 g/kg body weight. All animals behaved normally. No neurological or behavioral effects could be noted. No mortality was found up to 14 days study.

### Blood glucose level

**Figure 1:**
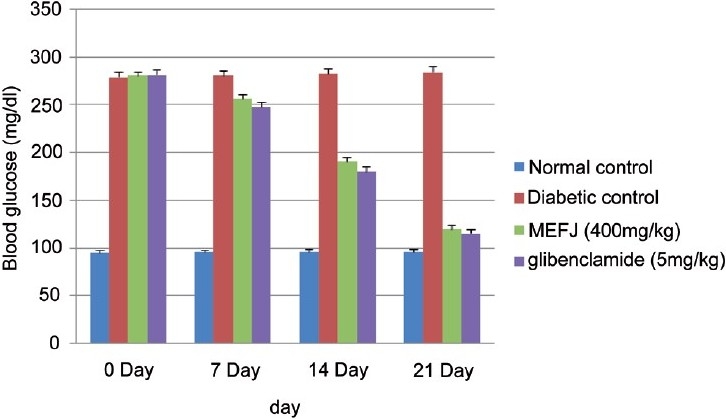
The effect of MEFJ and glibenclamide on blood glucose level in STZ-induced diabetic rats at various days (on 0 day, 7^th^ day, 14^th^ day, and 21^st^ day).

In STZ -induced diabetic rats, the blood glucose levels were in the range of 279–281 mg/dL, which were considered as severe diabetes. In the glibenclamide (5 mg/kg) and methanolic extract (400 mg/kg) treated groups, the peak values of blood sugar significantly decreased from 281.2 mg/ dL to 114.6 mg/dL and from 280.6 mg/dL to 119.2 mg/dL on the 21^st^ day, respectively [Table 1 and Figure 1]. Hence, in this study observations showed that the MEFJ reduced the blood glucose level in diabetic rats but values did not return to those of normal controls. Therefore, MEFJ possesses significant (*P* < 0.01) antidiabetic activity, when compared with diabetic control. There was a marked reduction in blood glucose level (in 21 days) in STZ -diabetic animals. This effect of the MEFJ (400 mg/kg) is nearly equal to, if not better than, that of glibenclamide (5 mg/kg) [Table 1 and Figure 1].

**Table 1:** Effect of MEFJ on blood glucose level in STZ-induced diabetic rats

*Groups*	*Blood glucose (mg/dL) in day*			
	*0*	*7*	*14*	*21*
Normal control (−)	94.2 ± 3.65	95 ± 2.7	95.8 ± 3.06	95.2 ± 2.37
Diabetic control (−)	279 ± 6.21	280.4 ± 6.01	282.6 ± 5.82	284.4 ± 5.25
Diabetic rats + MEFJ (400 mg/kg)	280.6 ± 5.16^ns^	256.6 ± 4.95**	190.4 ± 5.25**	119.2 ± 2.27**
Diabetic rats + glibenclamide (5 mg/kg)	281.2 ± 5.89^ns^	247.8 ± 5.76**	179.4 ± 6.48**	114.6 ± 3.17**

Values are mean ± SEM; n = 5 in each group except in diabetic control group where n = 4 because one animal died on the 8^th^ day.

ns *P* > 0.05 (non-significant)

** *P* < 0.01 (highly significant) when compared to diabetic control rats; MEFJ or glibenclamide was administered daily for 21 days. For glucose estimation, blood was collected just before the drug administration on the 0 day and 1 h after the drug administration on the 7^th^, 14^th^ day and 21^st^ day.

### Serum lipid profile

The effect of the MEFJ and glibenclamide, in the untreated diabetic rats’ serum levels of cholesterol and *TG* were significantly increased [Table 2]. These complications of diabetes were attenuated with the administration of the methanolic extract. The effects of the standard drug (glibenclamide) on serum TG and cholesterol in the diabetic rats were comparable to those of the herbal extract. Total cholesterol and TG were significantly elevated in diabetic group in comparison to control group. Administration of MEFJ for 21 days significantly reduced the serum levels of cholesterol and TG in comparison to diabetic control rats [Table 2].

**Table 2:** Effect of MEFJ on serum lipid profile in STZ-induced diabetic rats

*Groups*	*Cholesterol (mg/dL)*	*Triglycerides (mg/dL)*
Normal control	81.8 ± 3.69	74.6 ±5.19
Diabetic control	130 ± 8.47	120.2 ± 5.24
Diabetic rats + MEFJ	105.6 ± 6.06*	96.2 ± 4.38*
Diabetic rats + glibenclamide	87.2 ± 3.14**	84.4 ± 7.52**

Values are mean ± SEM; n = 5 in each group except in diabetic control group where n = 4 because one animal died on the 8^th^ day.

* *P* < 0.05 (significant)

** *P* < 0.01 (highly significant) when compared to diabetic control rats.

### Urine glucose and ketone

Urine analysis on day 0 showed the presence of glucose (+++) and ketone (trace) in the entire group, except normal control. However, on 21*st* day glucose and ketone traces were absent in MEFJ and glibenclamide treated groups while they were present in diabetic control group [Table 3].

**Table 3:** Effect of MEFJ on urine glucose and ketone in STZ-induced diabetic rats

*Groups*	*0 day*		*21 day*	
	*Glucose*	*Ketone*	*Glucose*	*Ketone*
Normal control	−	−	−	−
Diabetic control	+++	Trace	+++	Trace
Diabetic rats + MEFJ	+++	Trace	−	−
Diabetic rats + glibenclamide	+++	Trace	−	−

Glucose = absence of glucose, +++ = 1 g/dL; Ketone = absence of ketone, Trace = 5 mg/dL.

## Discussion

The various numbers of plants have been traditionally used to treat diabetes, and some have been proven to have hypoglycemic effects. These studies have identified that compounds such as polysaccharides,[16] flavonoids,[17] terpenoids and tannins,[18] and steroids[19] are responsible for antidiabetic effect. MEFJ also contains flavonoids, saponins and carbohydrate, steroids, tannins, and phenolic compounds. The observed hypoglycemic effects of this plant could have resulted from the combined activity of these compounds present in the extract.

Administration of STZ caused rapid destruction of pancreatic β-cells in rats, which led to impaired glucose stimulated insulin release and insulin resistance, both of which are marked feature of type II diabetes.[20] Oral hypoglycemic agents and insulin are currently available for treating DM. There is, however, a growing interest in herbal remedies due to the side effects associated with the existing drugs.[21] The present investigation indicates the hypoglycemic and also protective effects of MEFJ on serum lipid profile of STZ-diabetic rats. We have observed a significant (*P* < 0.01) decrease in blood glucose in MEFJ-treated diabetic rats, when compared with diabetic control rats. The possible mechanism of MEFJ on hypoglycemic action may be through potentiation of pancreatic secretion of insulin from β-cell of islets and/or due to enhanced transport of blood glucose to the peripheral tissue or by other mechanisms such as stimulation of glucose uptake by peripheral tissue, inhibition of endogenous glucose production or activation of gluconeogenesis in liver and muscles.[22]

Diabetes is associated with hyperlipidemia. It is well known that insulin activates enzyme lipoprotein lipase, which hydrolyzes triglyceride under normal conditions. Destruction of β-cells leads to depletion of plasma insulin, which results in hyperlipidemia. The significant control of plasma lipid levels suggests that the MEFJ may produce its action by improving insulin secretion.[23]

Diabetogenic agents significantly increase the cholesterol and TG levels. The abnormally high concentration of serum lipids in DM is mainly due to an increase in the mobilisation of free fatty acids from the peripheral fat depots, since insulin inhibits the hormone-sensitive lipase. The marked hyperlipidemia that characterises the diabetic state may, therefore, be regarded as a consequence of the uninhibited actions of lipolytic hormones on the fat depots. Excess of fatty acids in plasma produced by STZ promotes the liver conversion of some fatty acids to phospholipids and cholesterol. These two substances, along with excess of TG formed in the liver, may be discharged into lipoproteins in the blood. As a result, serum phospholipids are elevated.[24] Administration of MEFJ to diabetic rats improved the cholesterol and *TG* [Table 2]. This effect may be due to low activity of cholesterol biosynthesis enzymes and/or low level of lipolysis which are under the control of insulin.[25] Defects in carbohydrate metabolizing machinery and consistent efforts of the physiological system to correct the imbalance in carbohydrate metabolism place an overexertion on the endocrine system, which leads to the deterioration of endocrine control. Continuing deterioration of endocrine control exacerbates the metabolic disturbances and leads primarily to hyperglycemia.[15] The most significant findings of this study is that the MEFJ has shown beneficial effect not only on blood glucose, but also on glucose and ketone levels of urine in STZ -induced diabetic rats. Urine analysis on 0 day showed the presence of glucose and traces of ketone in the entire group except normal control. However, on 21^st^ day glucose and ketone traces were absent in MEFJ and glibenclamide-treated groups while they were present in diabetic control [Table 3]. Therefore, results obtained from this study are quite promising and comparable with glibenclamide, a standard drug used to treat DM.

The observations confirm that methanolic extract of the leaf and stem of the plant has antidiabetic activity and is also involved in correction of altered biological parameters. It also warrants further investigation to isolate and identify the hypoglycemic principles in this plant so as to elucidate their mode of action.
